# Serological and molecular detection of *Babesia caballi* and *Theileria equi* in Mexico: A prospective study

**DOI:** 10.1371/journal.pone.0264998

**Published:** 2022-03-08

**Authors:** Elizabeth Salinas-Estrella, Massaro W. Ueti, Vladislav A. Lobanov, Evelio Castillo-Payró, Amelia Lizcano-Mata, César Badilla, Francisco Martínez-Ibáñez, Juan Mosqueda

**Affiliations:** 1 Immunology and Vaccines Laboratory, Natural Sciences College, Autonomous University of Queretaro, Queretaro, Qro, Mexico; 2 Programa de Maestría y Doctorado en Ciencias de la Producción y de la Salud Animal, Facultad de Medicina Veterinaria y Zootecnia, Universidad Nacional Autónoma de México, Ciudad de México, México; 3 Department of Veterinary Microbiology and Pathology, Washington State University-USDA Animal Disease Research Unit, Pullman, Washington, United States of America; 4 Centre for Food-Borne and Animal Parasitology, Canadian Food Inspection Agency, Saskatoon, Saskatchewan, Canada; 5 Private Practice, Macultepec, Tabasco, México; 6 Departamento de Clínica de Grandes Especies, Facultad de Medicina Veterinaria y Zootecnia, Universidad Autónoma de Yucatán, Mérida, Yucatán, México; 7 Ingeniería en Producción Animal, Universidad Politécnica del Mar y la Sierra (UPMyS), La Cruz, Elota, Sinaloa, México; 8 Laboratorio de Taxonomía, Centro Nacional de Servicios de Constatación en Sanidad Animal Servicio Nacional de Sanidad Inocuidad y Calidad Agroalimentaria-Secretaría de Agricultura Ganadería Desarrollo Rural Pesca y Alimentación, Jiutepec, Morelos, México; University of Bari, ITALY

## Abstract

Equine piroplasmosis is a disease of horses, mules and donkeys, caused by the hemoprotozoans *Babesia caballi* and *Theileria equi* and transmitted by ticks of tropical and subtropical regions. Because the clinical signs are not specific, the diagnosis of equine piroplasmosis is difficult. In Mexico, where the environmental factors are conducive to the persistence of these pathogens, there is a lack of molecular studies to evaluate the occurrence of both parasites in horses. In the present study, matching serum and whole blood samples were obtained from 269 horses residing in 24 locations with tropical or subtropical climate and the presence of ticks. Testing of serum samples by ELISA demonstrated 55.7% seroprevalence of *B*. *caballi* and 68.4% prevalence of antibodies to *T*. *equi*. Blood samples analyzed with nPCR test were 7.8% positive to *B*. *caballi* and 78.8% positive to *T*. *equi*, while a duplex qPCR showed 15.24% positive samples to *B*. *caballi* and 59.11% to *T*. *equi*. From these results, 27 samples were sequenced for *T*. *equi* and 13 for *B*. *caballi*, confirming the presence of both horse parasites that cause equine piroplasmosis and suggesting that they are widespread in Mexico. This is the first study confirming the presence of *B*. *caballi* and *T*. *equi* in Mexico using both serological and molecular diagnostic methods. This study shows a high incidence of exposure to the etiological agents of equine piroplasmosis in horses in the studied areas.

## Introduction

Equine piroplasmosis (EP) is a disease that affects horses, mules and donkeys, and it is caused by the parasites *Babesia caballi* and *Theileria equi*. Equine piroplasmosis is distributed around the world, mainly in the tropical and subtropical regions and sometimes in temperate regions due to the appropriate conditions for the survival of ticks, which are the vectors of these parasites [1–5]. Both pathogens cause disease with similar clinical signs in the mammalian hosts such as anemia, thrombocytopenia, hemoglobinuria, jaundice, fever, lethargy, dyspnea, lack of appetite, mucosal congestion, sweat, inflamed abdomen and weakness in the acute infection. In contrast, in chronic cases, the equines can be observed with weight loss, splenomegaly, transitory fever and low exercise tolerance [5–7]. The diagnosis based on clinical signs is challenging because of the nonspecific symptoms. However, a definitive diagnosis can be made by microscopy during the acute phase of the disease and by serology using the complement fixation test (CFT), immunofluorescence assay (IFA) or the enzyme-linked immunosorbent assay (ELISA) [5, 8, 9]. Equine piroplasmosis can also be detected using polymerase chain reaction (PCR)-based assays [10, 11]): conventional PCR (cPCR) [12–14], nested PCR [15–17], quantitative PCR (qPCR) [18, 19], multiplex PCR [20] and duplex qPCR [21]. Five tick species capable of transmitting EP in Mexico are *Rhipicephalus microplus*, *Amblyomma mixtum (cajennense sensu lato)*, *Amblyomma imitator*, *Dermacentor albipictus* and *Anocentor nitens* [5]. These ticks can be distributed in at least 25.9% and up to 79.2% of Mexico’s continental surface, because of appropriate weather conditions present in several states. Most of these states are located in the west, south, southeast and east coast, and just a few states located in the central region have some areas with suitable climate conditions. According to The Food and Agricultural Organization of the United Nations (FAO), approximately 90% of the world’s equine population lives in piroplasmosis endemic areas [22], and in most of the developing countries, the equines that live in those areas are working animals. The OIE [23] has included equine piroplasmosis on the list of reportable diseases, and in Mexico, it is a mandatory monthly reported disease. The monitoring is made by the National Service for Agri-Food Health, Safety and Quality (SENASICA)–Secretary of Agriculture, Livestock, Rural Development, Fisheries and Food [24], using Giemsa and Wright staining, CFT, ELISA, and immunodiffusion. In Mexico *T*. *equi* and *B*. *caballi* have been reported in several states by microscopy [25], indirect fluorescence antibody test (IFAT) [8], conventional PCR [26] and nested PCR [27]. However, none of those studies used a combined detection of antibodies and DNA. The importance of studying this disease in the Mexican equine population lies in the fact that, according to the Mexico-European Union Agrofood Trade Balance, Mexico was the second supplier of horse meat (2,014 tons equivalent to 7,840 thousand euros) [28]. All of the above, coupled with the importance of horses as working animals that are indispensable in many places for the development of productive activities and the lack of molecular diagnostics and a vaccine for this disease, support the need for more extensive studies of the EP epidemiology in Mexico. Therefore, the aim of this work was to determine the presence of antibodies against both *B*. *caballi* and *T*. *equi* and the presence of the DNA of these parasites in equine blood samples from tick-endemic areas in six Mexican states.

## Materials and methods

This study was approved by the Institutional Subcommittee for the Care of Animals in Experimentation under the Mastery and Doctorate program in Sciences of Production and Animal Health of the National Autonomous University of Mexico (by the reception of the SICUAE format 26062015). In addition, we have obtained the owner’s consent for each of the horses involved in this study.

The formula to determine the sampling size when the total population is unknown was n = z^2^pq/d^2^ and it was used with a *z* (confidence level) of 95% and a *d* (estimated error) of 5%, then substituting the value of *p* (the probability of the event occurring) with an average value of 0.77 obtained from a review of other similar studies performed in different parts of the world [29, 30] and the value *q* (1-*p*) of 0.23. Thus, the resulting sample size was 272, and it was considered a minimum of 30 samples required to determine the presence of a disease in each state to ensure valid sampling [31]. The sampling was carried out using a non-probabilistic approach, as the owners had to agree to participate and give their consent to collect samples from their horses. The owners were contacted by veterinarians working in nearby areas at the time.

**Fig 1:**
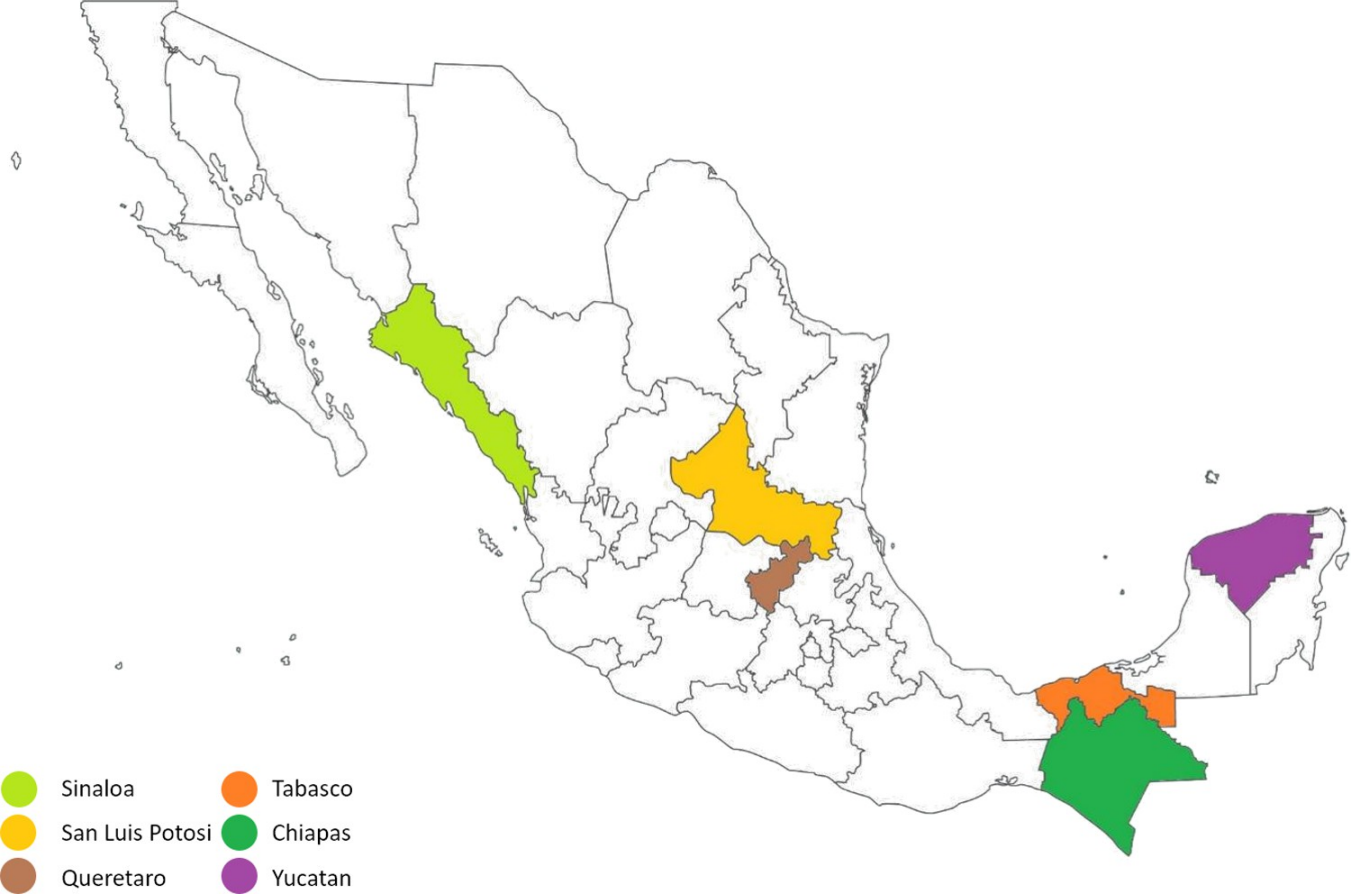
Sample collection. The samples were obtained in the states of Yucatan (purple), Tabasco (orange), Chiapas (green), Queretaro (brown), San Luis Potosi (yellow) and Sinaloa (light green).

Serum, blood and tick samples were collected form equines from six different tropical climate states in Mexico (Fig 1) and tested serologically by ELISA and molecularly by nPCR and duplex qPCR, respectively (see below).

The sampling was carried out between April 2015 and March 2016. The geographic and climate characteristics of the sampling areas are shown in Table 1. Blood samples were taken from the jugular vein and then centrifuged at 252 RCF in tubes without and with decoagulant, respectively. The blood and serum samples were stored at 4°C during their transportation to the laboratory and stored at -20°C until use upon arrival. Serum was collected in 1.5 ml tubes. From the blood samples, red blood cells were collected and stored in 1.5 ml tubes. The collected ticks were washed with distilled water and placed on absorbent paper to remove all external water droplets and kept at 4°C in Petri dishes before being sent to the Taxonomy Laboratory in the National Center for Animal Health Services (CENAPA), where they were analyzed according to the procedure and keys of this institution.

**Table 1:** Geographical and climatic characteristics of the sampling locations.

State	Location	Coordinates	Altitude ^a^	Average annual temperature ^b^	Average annual rainfall ^c^
**Chiapas**	Cintalapa	16° 39’ N & 93° 44’ W	540	24.5	800.0
	Tonalá	16° 06’ N & 93° 45’ W	60	27.1	1653.0
	Mapastepec	15° 26’ N & 92° 54’ W	50	22.2	2500.0
	Cacahoatán	14° 59’ N & 92° 10’ W	480	25.4	4120.0
	Tuxtla Gutiérrez	16° 45’ N & 93° 07’ W	600	26.5	2030.0
	Ocozocoautla	16° 45’ N & 93° 22’ W	820	23.4	951.0
	Playas de Catazajá	17° 44’ N & 92° 01’ W	10	26.4	2322.0
	Yajalón	17° 10’ N & 92° 20’ W	800	25.7	2120.0
	San Pablo Chalchihuitán	16° 58’ N & 92° 39’ W	1,450	22.5	2036.0
**Querétaro**	Arroyo Seco	21° 15’- 21° 35’ N/99° 25’ & 99° 47’ W	560	22.0	10.5
	Landa de Matamoros	21° 06’ y 21°27’ N 99° 03’ a 99° 22’ W	350	22.0	920.0
	Jalpan de Serra	21°40’- 21°05’N/99°06’- 99°32’ W	200	24.5	1500.0
**Tabasco**	Huimanguillo	17°19’ N 93°23’ W	29	26.9	2290.3
	Macultepec	18°20’ N 93°15’ W	10	33.6	2237.0
	Nacajuca	18°09’ N 93°01’ W	10	26.4	1707.0
**Yucatán**	Tzucacab	19° 38’ & 20° 09’ N 88° 59’ & 89° 14’ W	36	25.8	108.4
	Sucilá	21°07’- 21°14’ N/ 88° 16’-88° 25’ W	12	26.4	70.1
	Buctzotz	21°06’ - 21°25" N / 88°21”- 88°51" W	7	26.3	469.0
	Tizimín	07°58’ N/88°09’04’’ W	15	25.8	1,084
	Muna	20°24’-20°35’ N/ 89°37’-89°47’ W	29	25.5	69.7
	Temozón	20° 48’-20° 57’ N /87° 47’-88° 16’ W	22	25.8	82.0
**Sinaloa**	Culiacán	24°02"- 25°14"N/106°56"-107°50"W	53	24.0	658.0
**San Luis Potosí**	Ciudad Valles	21° 59"N / 99°01"W	70	24.5	1400.0
	Tamuín	21°46"- 22°24"N/98°24"- 98° 27" W	20	25.8	883.0

^a^ The altitude of the locations is expressed as meters above the sea level (masl).

^b^ The temperature is expressed in Celcius degrees.

^c^ The average rainfall is expressed as millimeters per year.

Source: INEGI [28]

All serum samples were tested for the presence of antibodies to *B*. *caballi* and *T*. *equi* by cELISA using kits manufactured by VMRD (Pullman, WA, USA). The assays were performed in strict accordance with the manufacturer’s protocols, whereby a test sample producing ≥ 40% inhibition was considered positive. Optical density values were obtained using an iMark™ Microplate Absorbance Reader (BioRad, Hercules, CA, USA) and the Microplate Manager 6™ software.

Genomic DNA was extracted and purified from the blood samples, the Illustra Blood Genomic Prep mini spin kit™ (GE Health Care Life Sciences, USA) was used. The protocol was carried out according to the user manual with the only modification of using 150 μl of red blood cells instead of 300 μl of whole blood. All the DNA extracted samples were quantified using a NanoDrop 2000™ (Thermo Fischer Scientific, Waltham, MA, USA).

The *T*. *equi* nPCR for amplification of an *ema1* gene fragment utilized primers and thermocycling protocol published by Ueti *et al*. [32], while the nPCR for *B*. *caballi* (for amplification of a *rap1* gene fragment) was based on the protocol published by Schwint *et al*. [17]. Specificity was tested before testing the samples, using DNA purified from *Anaplasma marginale*, *A*. *ovis* and *T*. *equi* and *B*. *caballi* with the opposite of each parasite specific primers respectively. Positive controls were genomic DNA of both equine parasites, respectively, obtained from Washington State University. The sensitivity of nPCR for *T*. *equi* was <10 parasites and a single parasite for *B*. *caballi* [17, 33].

A second extraction of total DNA from the 269 samples followed by testing with the duplex qPCR that detects both these parasites in a single reaction was performed at the Center for Food-borne and Animal Parasitology, Canadian Food Inspection Agency, as previously reported [21]. This assay amplifies at least 10 copies of the *ema1* gene of *T*. *equi* while the sensitivity for *B*. *caballi* was 4 x 10^−6^% infected cells. Negative controls consisting of nuclease-free water and DNA extracted from the blood of an EP-free horse (Western College of Veterinary Medicine, University of Saskatchewan, Saskatoon, Canada) were included on each qPCR plate. Preparations of DNA extracted from stabilates of blood from horses infected with either *T*. *equi* or *B*. *caballi* (NVSL/USDA) were used as positive controls.

To obtain the nucleotide sequences from detected piroplasms for analysis, 13 *B*. *caballi* nPCR-positive and 27 *T*. *equi* nPCR-positive samples were amplified by conventional PCRs with primers flanking full-length *rap1* (*B*. *caballi*) and *ema1* (*T*. *equi*) genes. The primers for the *rap1* gene were (Forward) 5’-ATGAGGTGTTCTGCGAGTT-3’ and (Reverse) 5’-GAGAGAGGCTTCATAGTTGTC-3’, whereas primers for the *ema1* gene were (Forward) 5’-ATGATTTCCAAATCCTTTGCT-3’ and (Reverse) 5’-GTAAAATAGAGTAGAGAATGCAATGG-3’. The amplified products were cloned into a plasmid vector using the TOPO™ system (Thermo Fisher Scientific). The cloned fragments were submitted for sequencing with M13 forward and reverse universal primers. The obtained forward and reverse reads were corrected and assembled into contigs using Clone Manager Professional 9 software (Sci-Ed Software). Amino acid sequences were deduced, and non-unique duplicate sequences were removed using Geneious 11.1.5 (Biomatters). The remaining unique sequences were aligned with similar sequences available in GenBank using the MAFFT plugin. After maskin gaps in the alignment, phylogenetic trees were built using Geneious Tree Builder by Neighbor-Joining method with the bootstrap number of replicates set to 1,000.

## Results

**Fig 2:**
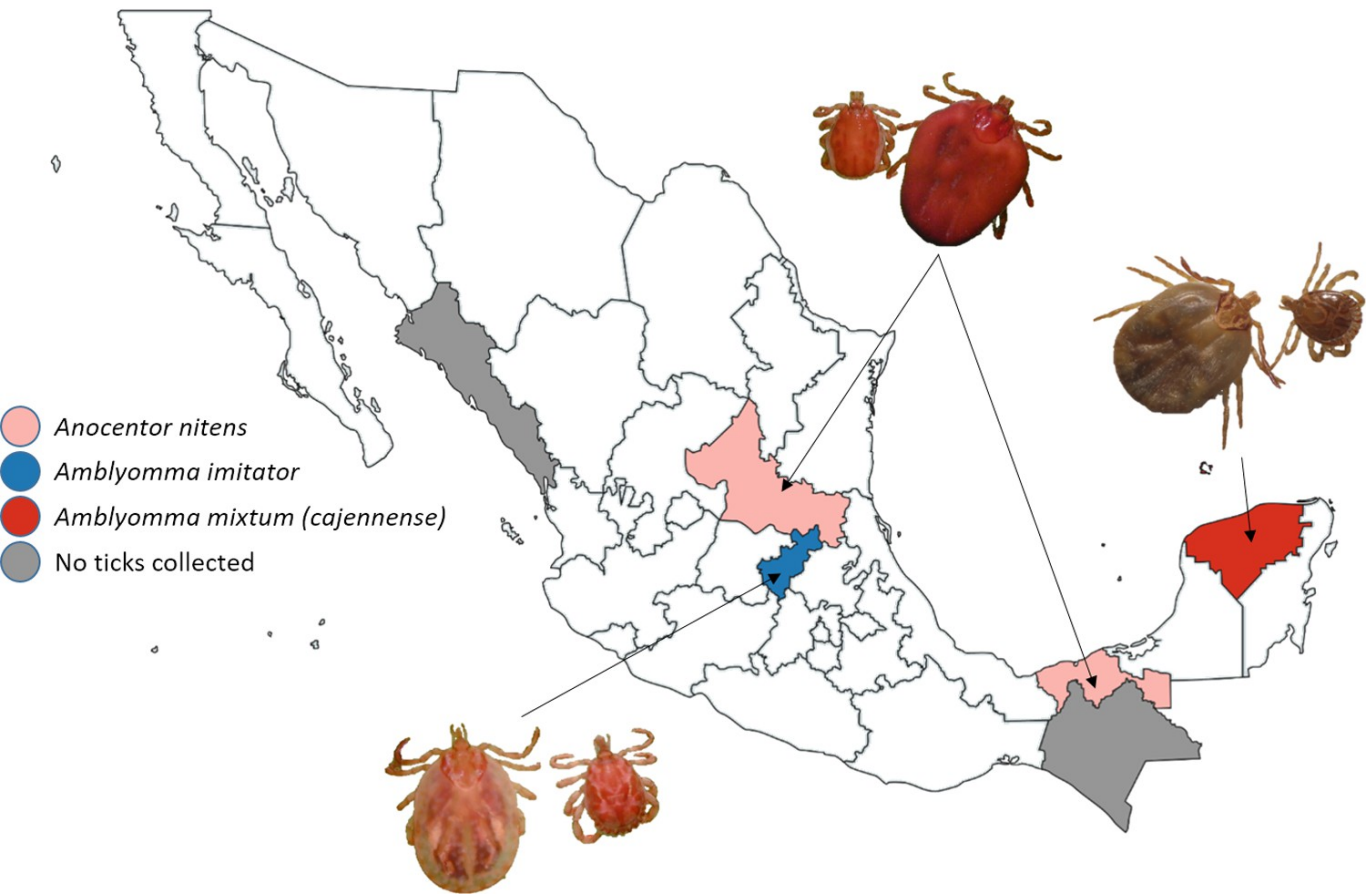
Taxonomy analysis results. Ticks from Tabasco and San Luis Potosi (pink). Ticks from Queretaro (blue) and ticks from Yucatan (red). No ticks were found on equines from Sinaloa and Chiapas (grey).

Sample collections were performed on 269 horses in 24 different locations; 30 samples were obtained from Queretaro state, 33 samples from Tabasco, 77 samples from Chiapas, 61 samples from Yucatan, 34 samples from San Luis Potosi and 34 samples from Sinaloa (Fig 1). The locations in which the horses were sampled were the result of the location of the owners willing to participate in the sampling, wich was therefore performed by convenience (Table 1). Ticks were found on horses from the states of Yucatan, Queretaro, Tabasco and San Luis Potosi. There were no detectable ticks on equines from Sinaloa and Chiapas at the moment of the sample collection. The ticks sent for analysis were identified as *Anocentor nitens*, *Amblyomma imitator* and *Amblyomma mixtum (cajennense)* (Fig 2).

**Fig 3:**
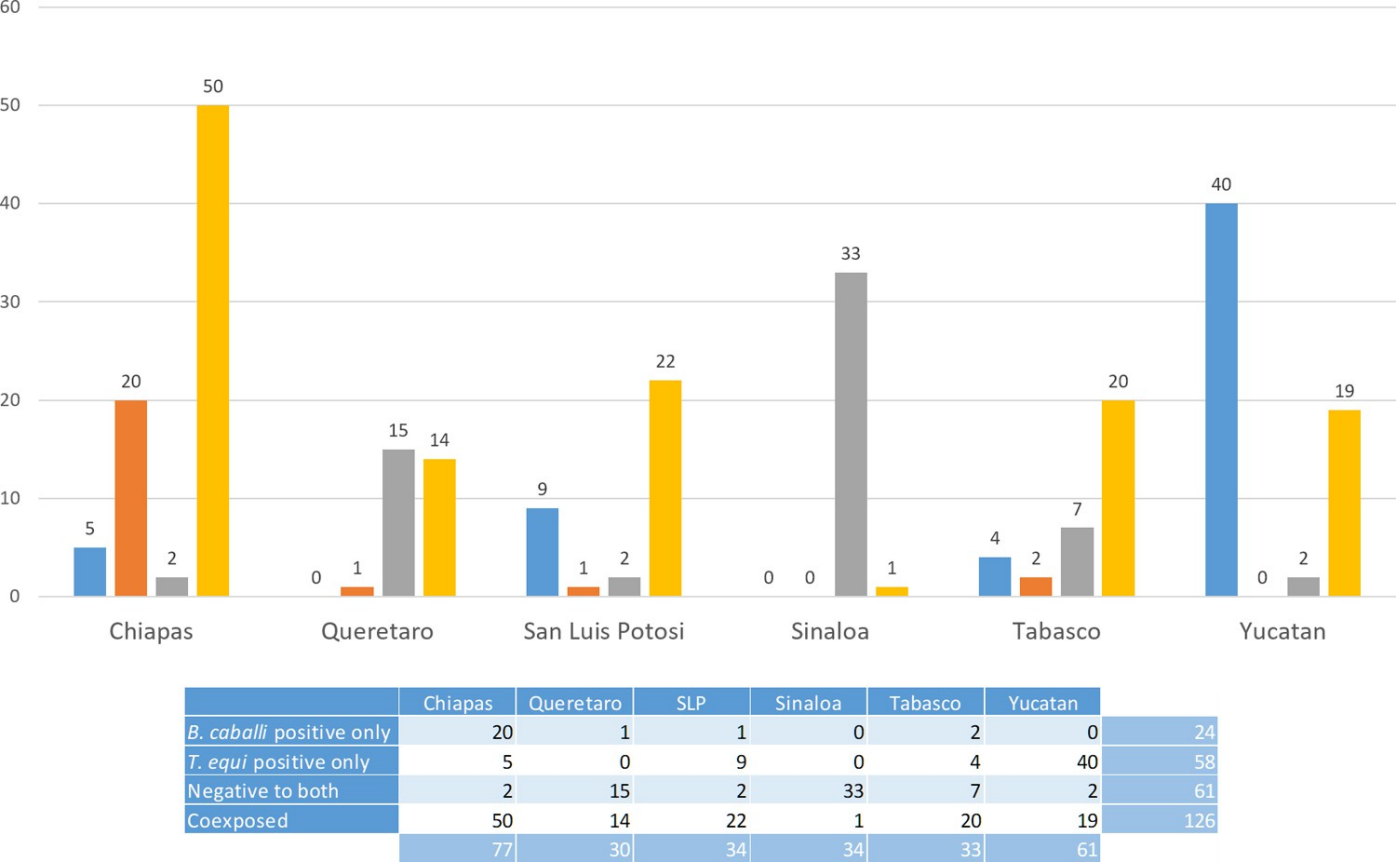
Antibody detection by cELISA. Positive samples to *T*. *equi* (blue bars). Positive samples to *B*. *caballi* (orange bars). Negative samples (grey bars). Coexposed (yellow bars). The table section shows a summary of the results.

**Fig 4:**
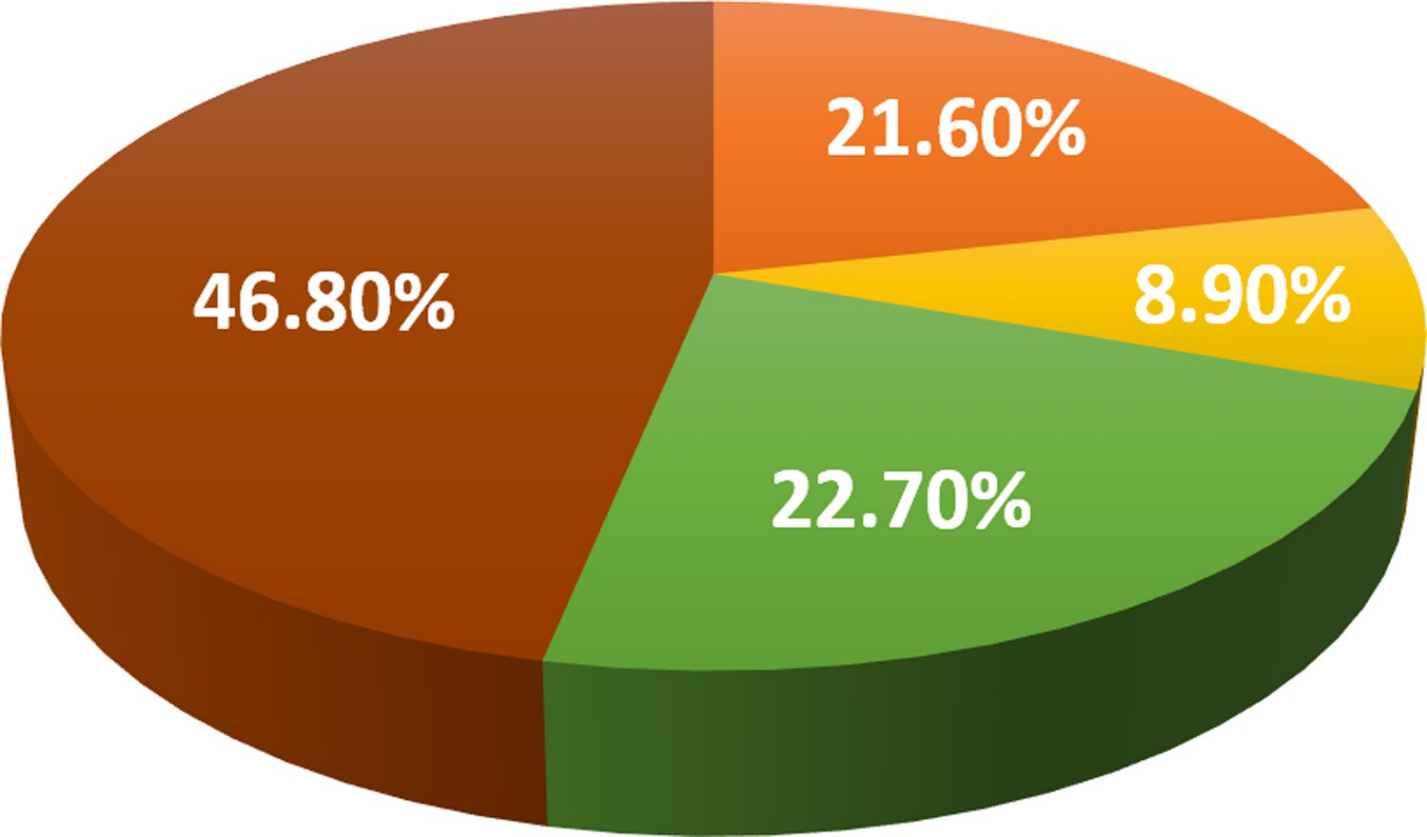
Antibody detection percentages for cELISA. Positive samples to *T*. *equi* (orange). Positive samples to *B*. *caballi* (yellow). Double-negative (green). Double-positive (brown).

Serological analysis showed a high number of animals were coexposed to both pathogens (Fig 3). In summary, from a total of 269 samples (100%), 21.6% were positive for only *T*. *equi*, 8.9% were positive for only *B*. *caballi*, 22.7% were negative for both parasites, and 46.8% revealed coexposure (Fig 4). Hence, the total number of positive samples to antibodies against *T*. *equi* was 184 (68.4%), whereas 150 (55.7%) were positive to antibodies against *B*. *caballi*.

**Fig 5:**
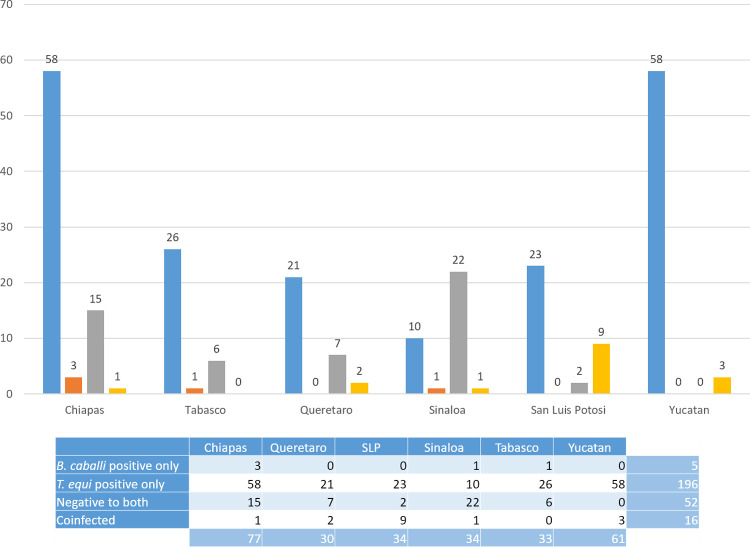
Molecular detection by nPCR. Positive samples to *T*. *equi* (blue bars). Positive samples to *B*. *caballi* (orange bars). Negative samples (grey bars). Coinfected samples (yellow bars). The table section shows a summary of the results.

**Fig 6:**
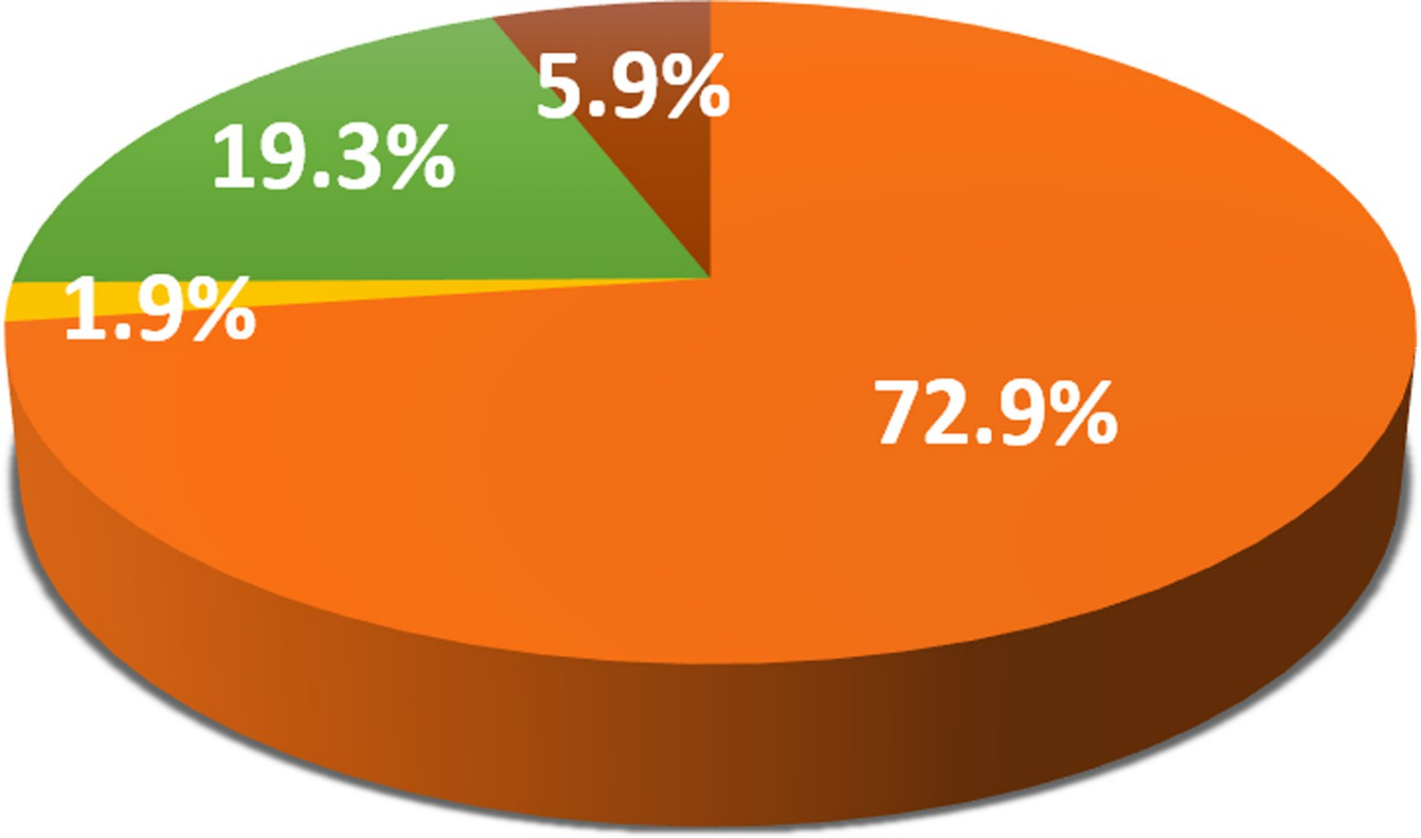
Detection percentages for nPCR. Positive samples to *T*. *equi* (orange), positive samples to *B*. *caballi* (yellow). Double-negative (green). Double-positive (brown).

Out of 269 samples tested for *B*. *caballi* and *T*. *equi* by nPCR, the majority were found positive to *T*. *equi* (Fig 5). In summary, 72.9% were positive for *T*. *equi*, 1.9% were positive for *B*. *caballi*, 19.3% were negative for both parasites and 5.9% were co-infected (Fig 6). Overall, the total numbers of DNA samples positive for *T*. *equi* and *B*. *caballi* were 212 (78.8%) and 21 (7.8%), respectively.

**Fig 7:**
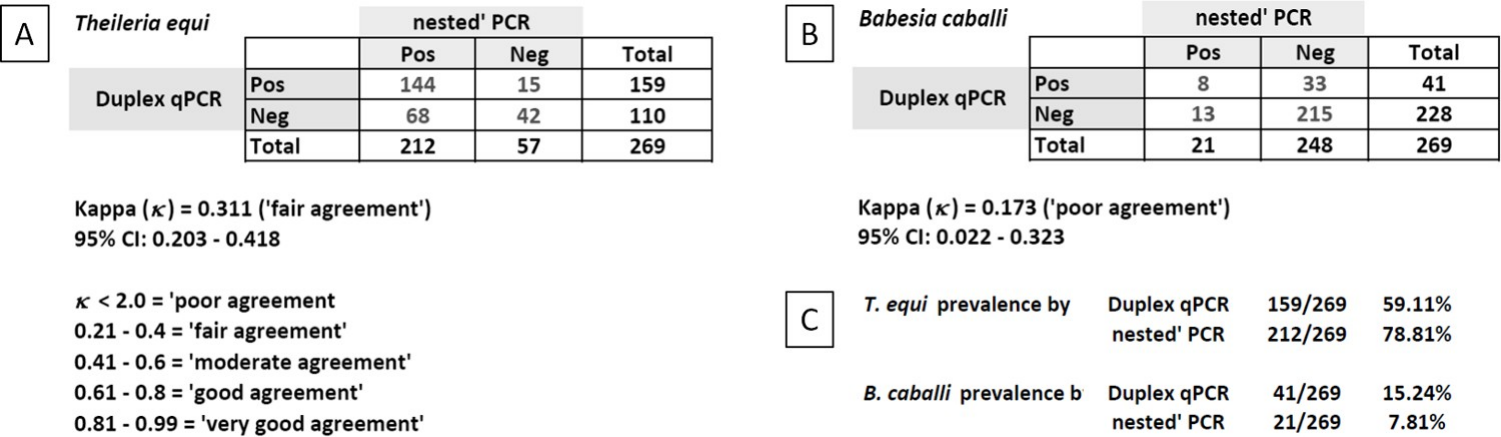
Kappa statistic calculations for the test results generated by the molecular assays used in this study. A) 2×2 contingency table for *T*. *equi*. B) 2×2 contingency table for *B*. *caballi*. C) Infection prevalences calculated using the nPCR and duplex qPCR test results.

Finally, the duplex qPCR assay was performed on all animals sampled to compare with the nPCR results. Results of qPCR showed a *T*. *equi* prevalence of 59.11% (159/269) and *B*. *caballi* prevalence of 15.24% (41/269). The Kappa statistics for comparing the test results of these two molecular assays were 0.311 (fair agreement) and 0.173 (poor agreement), respectively (Fig 7).

**Fig 8:**
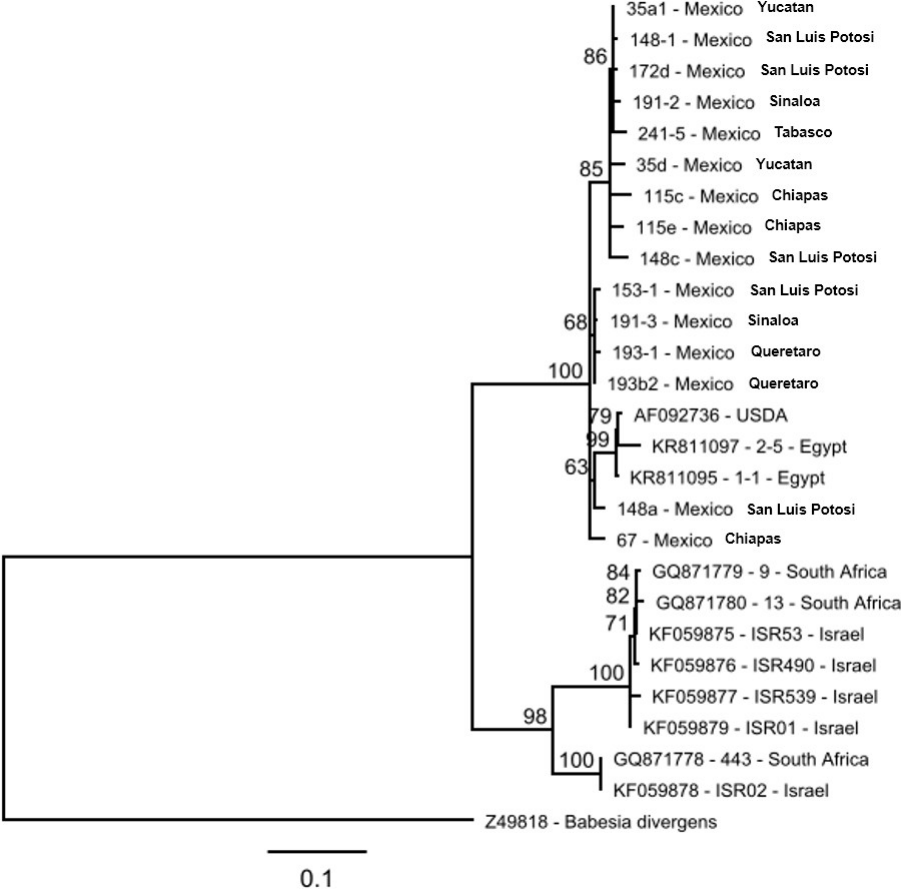
Phylogenetic tree constructed using RAP1 amino acid sequences generated in this study and similar sequences deposited in GenBank (sequence names start with an accession number). Translated Z49818 sequence of the *B*. *divergens rap1* gene served as an outgroup. Bootstrap support values over 50 are shown on the nodes.

Based on the nPCR results, 27 *T*. *equi-*positive and 13 *B*. *caballi-*positive samples were selected and amplified by conventional PCR. The amplicons were cloned using the TOPO® system, and the cloned PCR products were sequenced to obtain full-length *ema1* of *T*. *equi* and full-length *rap1* of *B*. *caballi*. Of 84 *ema1* and 26 *rap1* clones sequenced, 80 and 19 quality assemblies were generated, respectively. To reduce the number of *ema1* sequences, non-unique duplicate sequences were removed, resulting in 55 unique sequences used in the downstream analysis. The alignment of 19 *rap1* nucleotide sequences demonstrated identities ranging from 97.1 to 99.9%. The range of identity values among these sequences transcribed was from 91.6 to 100%. The identity value ranges between the 55 Mexican *ema1* sequences were 93 to 100% at the nucleotide and 90.4 to 100% at the amino acid level. Phylogenetic analysis of transcribed truncated (N-terminal 330 amino acids) RAP1 sequences demonstrated that all Mexican sequences clustered together. This cluster also included RAP1 sequences of the USDA strain (AF092736) and Egyptian isolates of *B*. *caballi* (KR811097 and KR811085) (Fig 8). The RAP1 sequences of *B*. *caballi* isolates from South Africa and Israel were the most distant and separated into two subclusters as previously described [34]. A notable feature was the diversity of cloned RAP1 sequences originating from an individual infected horse (e.g., clones 148–1, 148c and 148a).

**Fig 9:**
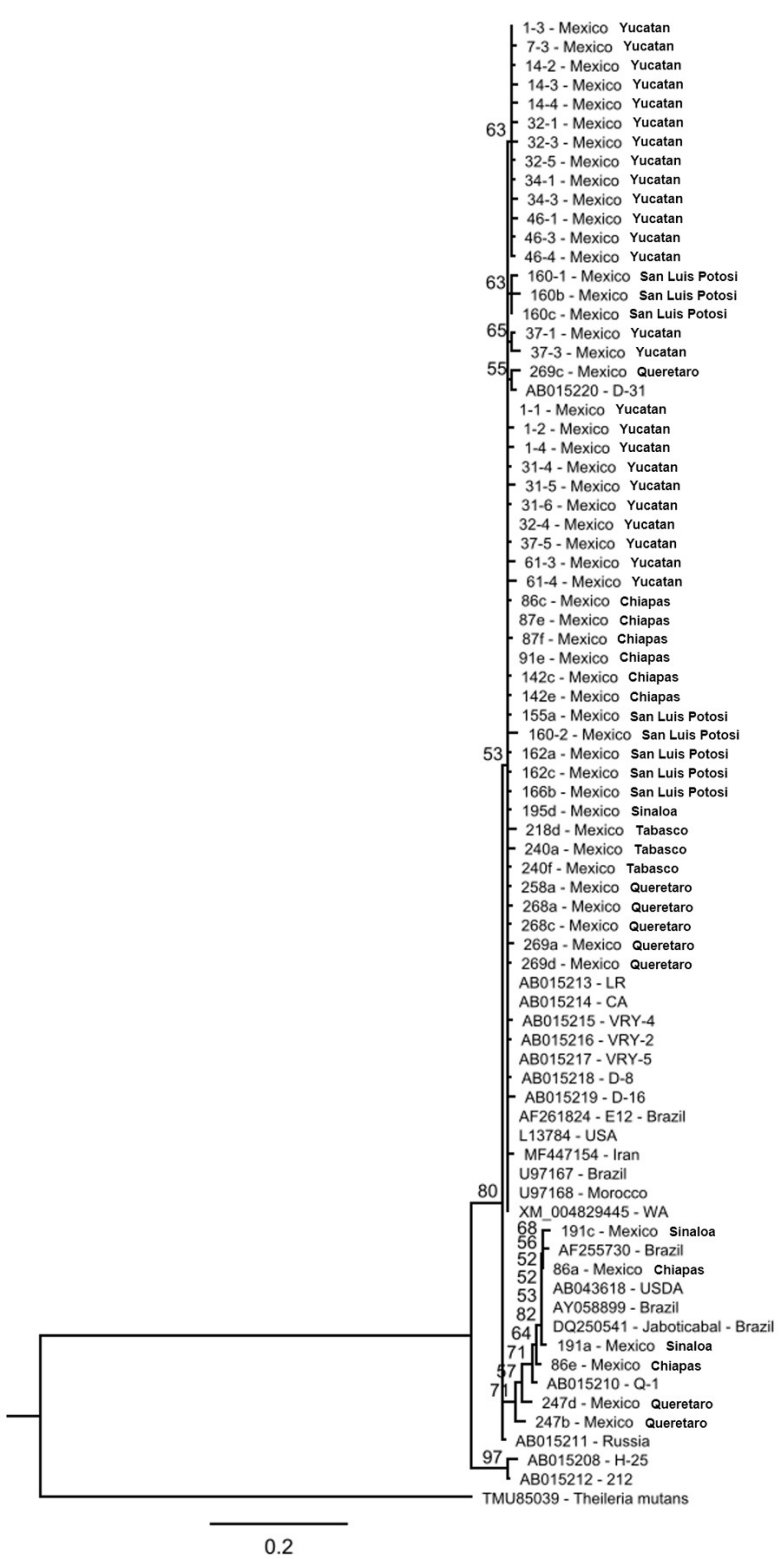
Phylogenetic tree constructed using EMA1 amino acid sequences generated in this study and similar sequences deposited in GenBank (sequence names start with an accession number). Translated TMU85039 sequence of the *T*. *mutans* homologue served as an outgroup. Bootstrap support values over 50 are shown on the nodes.

The analysis of transcribed EMA1 sequences demonstrated a high level of identity among the sequences analyzed (Fig 9). The only cluster with sufficient nodal support for separation was represented by two sequences (AB015208 and AB015212) deposited by researchers from Japan.

## Discussion

This study highlighted the risk of *B*. *caballi* and *T*. *equi* infection in horses from Mexico, supported also by the collection of tick species indicated as vectors for these pathogens. Indeed, the taxonomic analysis of the ticks collected here revealed the presence of *Amblyomma imitator* in Queretaro, *Amblyomma mixtum (cajennense)* in Yucatan and *Dermacentor (Anocentor) nitens* at San Luis Potosí and Tabasco. Wise *et al*. [35] pointed to *Amblyomma cajennense (sensu lato)* and *Rhipicephalus microplus* as vectors of *T*. *equi*, while *Dermacentor nitens*, *D*. *albipictus* and *D*. *variabilis* as tick species capable of transmitting *B*. *caballi* in North America. Thus, the results are coherent with the described before about the presence of these tick species as possible vectors of these piroplasm species. However, in this study, no experiments with the ticks were performed to test and confirm the transmission of the parasites or their presence in the tick tissues.

Differences in the percentage of positive samples were found compared with the reported at the National Diagnostics Laboratory (NDL) from SENASICA. From 2010 to 2016, 6,790 samples from all over the country were tested for EP by NDL, of which 643 (9.46%) were positive. Particularly, in the same period, 1,067 samples from the same six states as those included in this study were analyzed for EP at NDL, resulting in 177 (16.78%) positive samples. This is in significant contrast with the present results; nevertheless, the diagnostic methods are not the same, and the techniques used in this study, the cELISA, nPCR tests and duplex qPCR, have proven to detect even carrier animals [17, 21, 32, 36, 37].

The percentage of positive samples for *B*. *caballi* was higher (55.7%) when using cELISA compared to nPCR (7.8%). This outcome was expected and may have various explanations: since parasitemia levels fluctuate and there were only single sample collections from each individual animal, some samples could have parasitemias below the nPCR detection levels. On the other hand, specific antibodies persist for a prolonged time after the clearance of *B*. *caballi* from the mammalian host [4], and this species is capable of adhering to capillary endothelium, with very low numbers in circulating blood [38]. These results are similar to those reported by Rosales *et al*. [30], with 23.2% positive samples to antibodies against *B*. *caballi* and just 4.4% of DNA positive samples. In the case of *T*. *equi*, the proportion of seropositive samples (68.4%) was less than the nPCR-positive samples (78.8%). This could be due to early infections with parasites detectable in blood in the absence of serum antibodies in a proportion of animals, or, in rare cases, could have happened due to the higher severity of the disease caused by *T*. *equi* that is described by some authors. Also could involve a minor amount of protective antibodies, lack of memory antibodies production or maybe a state of immune tolerance against this parasite due that has been reported as uncommon to clear from the host [16] and to remain present in circulating blood at detectable levels (despite fluctuating parasitemia levels) throughout the infection stages [21, 38]. Rosales *et al*. [30] reported 14% positive samples to serology and 61.8% positive samples to the presence of *T*. *equi* DNA. On the other hand, García-Bocanegra *et al*. [29] showed a higher seropositivity to *T*. *equi* (50.3%) than to *B*. *caballi* (11.4%). These two studies showed trends in the results, both serology and molecular testing, similar to the results obtained in this work.

This study showed a comparatively low agreement between the nPCR and duplex qPCR test results for both piroplasm species. A higher proportion of these horses tested positive for *B*. *caballi* by the duplex qPCR. It could be due to a higher sensitivity of duplex qPCR compared to the *B*. *caballi*-specific nPCR, as the former assay targets a multi-copy gene (i.e., 18S rRNA) in the *B*. *caballi* genome. In addition, there is the possibility that a better-quality DNA has been obtained in the second extraction and when used for duplex qPCR, a better amplification reaction has been allowed, which explains results with a higher prevalence. In the case of *T*. *equi*, fewer samples were positive for duplex qPCR amplification than for nPCR. Both assays have similar sensitivities, which may suggest that some of the samples could be contaminated when performing nPCR, which is a disadvantage of this technique. The duplex qPCR was previously extensively validated using samples from a representative number of horses from Brazil [21]. In that study, a high level of agreement between the duplex qPCR and two other earlier published singleplex qPCR assays for *B*. *caballi* and *T*. *equi* has been revealed. Real-time PCR does not require post-amplification processing of the amplicons that renders it less susceptible to carry-over contamination. This may be a possible explanation for the differences found, since nested PCR requires the manipulation of amplicons from the first reaction and this point represents a risk of contamination of the genetic material in the second reaction. It may be preferable to apply qPCR in future epidemiological surveys for EP in laboratories with a capacity for this technique.

Analysis of the DNA sequences obtained showed a high percentage of identity with the sequences reported at NCBI for both parasites, indicating that the positive samples were infected with *B*. *caballi* or *T*. *equi* in a similar manner as reported by Romero-Salas *et al*. [26] and Mahmoud *et al*. [39] with the identity analysis of DNA positive to *T*. *equi* and *B*. *caballi* respectively.

The importance of these results relies on the possibility to explore more about the transmission mechanisms, because this disease can be undetected once the equines pass the acute stage and, mostly in the case of *T*. *equi* infections, become asymptomatic carriers [38, 40]. This is a major problem due to the continuous movement of these animals from one place to another because of the sports competitions, the property changes that may result in the loss of past clinic information in some cases, in addition to the lack of care in the handling of medicines (subdosage, incomplete treatments or the share of needles) by the non-veterinary caregivers in some places and the lack of information about the presence of this disease in the country which drives to many owners and even veterinarians to forgo testing for this disease. Therefore, it is necessary to do an efficient and representative epidemiological study of this disease in the Mexican territory to determine the regions that present these pathogens; it is also important to repeat the tests in serologically positive and molecularly negative animals and vice versa. Besides, a transmission study of this disease to the equines by the different tick species is needed, whereby interinstitutional cooperation is vital to obtain more information and adequate feedback for a complete analysis and establishment of preventive and control measures, and the subsequent capacitation to workers, horse caretakers and veterinarians prioritizing early diagnosis to avoid dispersion.

## Conclusions

Out of 269 equine serum and blood samples obtained from six Mexican states 55.7% were positive to antibodies against *B*. *caballi*, 7.8% were positive by *B*. *caballi*-specific nPCR, and 15.24% were positive for *B*. *caballi* by duplex qPCR. Meanwhile, for *T*. *equi*, 68.4% of the samples were positive to the presence of antibodies, 78.8% were positive by nPCR, and 59.11% were positive by duplex qPCR. Identity analysis of several sequences confirmed infection with *B*. *caballi* or *T*. *equi* in nPCR-positive samples. In addition, tick species *Amblyomma mixtum*, *A*. *imitator* and *Anocentor nitens* are present on equines in warm climate regions, as the literature has reported. These data lead us to conclude that *B*. *caballi* and *T*. *equi* are present in asymptomatic equines in Mexico. Those chronically infected animals represent a reservoir for the potential spread of the disease, which has a wide distribution due to the presence of the biological vectors as well as the movement of subclinically infected equines.

## References

[1] Nuttall GHF, Strickland C. On the occurrence of two species of parasites in equine “piroplasmosis” or “biliary fever”. Parasitol. 1912; 5:65–96.

[2] De Waal DT. Equine piroplasmosis: a review. British Vet J. 1992; 148:6–14. doi: 10.1016/0007-1935(92)90061-5 1551016

[3] Mehlhorn H, Schein E. Redescription of Babesia equi (Laveran, 1901) as *Theileria equi*. Parasitol Res 1998; 84(6):467–475. doi: 10.1007/s004360050431 9660136

[4] Schwint ON, Ueti MW, Palmer GH, Kappmeyer LS, Hines MT, Cordes RT, et al. Imidocarb dipropionate clears persistent *Babesia caballi* infection with elimination of transmission potential. Antimicrob Agents Chemother. 2009; 53(10):4327–32. doi: 10.1128/AAC.00404-09 19620328 PMC2764191

[5] Scoles AG, Ueti WM. Vector ecology of equine piroplasmosis, Annu Rev Entomol. 2015; 60:561–80. doi: 10.1146/annurev-ento-010814-021110 25564746

[6] Rothschild CM, Knowles DP. Equine piroplasmosis, In Equine Infectious Diseases, Ed. Sellon DC, Long Mt. Saunders Elsevier, St. Louis, MO. 2007. p. 465–473.

[7] Rovid A, Allen J, Gaylon J, Lofstedt J, Victoria M. Emerging and exotic diseases of animals. First edition. Iowa, USA. 2010. p. 256–258.

[8] Cantú-Martínez MA, Segura-Correa JC, Silva-Páez ML, Avalos-Ramírez R, Wagner GG. Prevalence of antibodies to *Theileria equi* and *Babesia caballi* in horses from Northeastern Mexico. J Parasitol. 2012; 98(4): 869–870. doi: 10.1645/GE-3064.1 22339765

[9] Mosqueda J, Olvera-Ramírez A, Aguilar-Tipacamú G, Cantó GJ. Current Advances in Detection and Treatment of Babesiosis. Curr Med Chem. 2012; 19:1504–1518. doi: 10.2174/092986712799828355 22360483 PMC3355466

[10] Sahagún-Ruiz A, Waghela SA, Wagner GG. Cloning and expression of a *Babesia caballi* protein from a genomic library in Lambda Zap II. International Information System for the Agricultural Science and Technology. Vet Mex. 1995. ISSN: 0301-5092.

[11] Rampersad J, Cesar E, Campbell MD, Samlal M, Ammons D. A field evaluation of PCR for the routine detection of *Babesia equi* in horses. Vet Parasitol. 2003; 114:81–87. doi: 10.1016/s0304-4017(03)00129-8 12781470

[12] Bashiruddin JB, Camma C, Rebelo E. Molecular detection of *Babesia equi* and *Babesia caballi* in horse blood by PCR amplification of part of the 16S rRNA gene. Vet Parasitol. 1999; 84:75–83. doi: 10.1016/s0304-4017(99)00049-7 10435792

[13] Vargas D, Bonet R, Oliva P, Campano S. Implementación de la técnica de PCR en la identificación de *Babesia* ssp en equinos. Parasitol Latinoam. 2004; 59:179–182.

[14] Alhassan A, Iseki H, Kim C, Yokoyama N, Igarashi I. Comparison of polymerase chain reaction methods for the detection of *Theileria equi* infection using whole blood compared with pre-extracted DNA samples as PCR templates. Trop Anim Health Prod. 2007; 39:369–374. doi: 10.1007/s11250-007-9025-1 17944307

[15] Nicolaiewsky TB, Richter MF, Lunge VR, Cunha CW, Delagostin O, Ikuta N, et al. Detection of *Babesia equi* (Laveran, 1901) by nested polymerase chain reaction. Vet Parasitol. 2001. 31;101(1):9–21. doi: 10.1016/s0304-4017(01)00471-x 11587829

[16] Ueti MW, Palmer GH, Kappmeyer LS, Statdfield M, Scoles GA, Knowles DP. Ability of the vector tick *Boophilus microplus* to acquire and transmit *Babesia equi* following feeding on chronically infected horses with low-level parasitemia. J Clin Microbiol. 2005; Aug. 3755–3759. doi: 10.1128/JCM.43.8.3755-3759.2005 16081906 PMC1233951

[17] Schwint ON, Knowles DP, Ueti MW, Kappmeyer LS, Scoles GA. Transmission of *Babesia caballi* by *Dermacentor nitens* (Acari: Ixodidae) is restricted to one generation in the absence of alimentary reinfection on a susceptible equine host. J Med Entomol. 2008;45(6):1152–1155. doi: 10.1603/0022-2585(2008)45[1152:tobcbd]2.0.co;2 19058641

[18] Kim CM, Blanco LB, Alhassan A, Iseki H, Yokoyama N, Xuan X, et al. Diagnostic real-time PCR assay for the quantitative detection of *Theileria equi* from equine blood samples. Vet Parasitol. 2008; 151:158–163. doi: 10.1016/j.vetpar.2007.10.023 18077095

[19] Kappmeyer LS, Thiagarajan M, Herndon DR, Ramsay JD, Caler E, Djikeng A, et al. Comparative genomic analysis and phylogenetic position of *Theileria equi*. BMC Genomics. 2012; 13, 603. doi: 10.1186/1471-2164-13-603 23137308 PMC3505731

[20] Alhassan A, Pumidonming W, Okamura M, Hirata H, Battsetseg B, Fujisaki K, et al. Development of a single-round and multiplex PCR method for the simultaneous detection of *Babesia caballi* and *Babesia equi* in horse blood. Vet Parasitol. 2005; 129:43–9. doi: 10.1016/j.vetpar.2004.12.018 15817201

[21] Lobanov VA, Peckle M, Massard CL, Scandrett WB, Gajadhar AA. Development and validation of a duplex real-time PCR assay for the diagnosis of equine piroplasmosis. Parasites Vectors. 2018; 11:125. doi: 10.1186/s13071-018-2751-6 29499748 PMC5834856

[22] FAO. Food and agriculture data. (Consulted in october 2021). https://www.fao.org/faostat/en.

[23] OIE. Terrestrial Animal Health Code, Chapter 1.2 Criteria for the inclusion of diseases, infections and infestations in the OIE list. (Consulted in october 2021) https://www.oie.int/en/what-we-do/standards/codes-and-manuals/terrestrial-code-online-access/?id=169&L=1&htmfile=chapitre_criteria_diseases.htm.

[24] SAGARPA. Acuerdo mediante el cual se enlistan las enfermedades y plagas de los animales, exóticas y endémicas de notificación obligatoria en los Estados Unidos Mexicanos. 2018. (Consulted in october 2021). http://dof.gob.mx/nota_detalle.php?codigo=5545304&fecha=29/11/2018.

[25] Rodríguez-Vivas RI, Cob-Galera LA, Domínguez-Alpizar JL. Hemoparásitos en bovinos, caninos y equinos diagnosticados en el laboratorio de Parasitología de la Facultad de Medicina Veterinaria y Zootecnia de la Universidad Autónoma de Yucatán (1984–1999). Rev Biomed. 2000; 11:277–282.

[26] Romero-Salas D, Solis-Cortés M, Zazueta-Islas HM, Flores-Vásquez F, Cruz-Romero A, Aguilar-Domínguez M, et al. Molecular detection of Theileria equi in horses from Veracruz, Mexico. Ticks Tick Borne Dis. 2021; 12(3):101671. doi: 10.1016/j.ttbdis.2021.101671 33545504

[27] Ayala-Valdovinos MA, Lemus-Flores C, Galindo-García J, Bañuelos-Pineda J, Rodríguez-Carpena JG, Sánchez-Chiprés D, et al. Diagnosis and prevalence of *Theileria equi* horses in western Mexico by nested PCR. Parasitol Int. 2017; 66(1):821–824. doi: 10.1016/j.parint.2016.09.011 27671686

[28] INEGI. Anuario estadístico y geográfico de los Estados Unidos Mexicanos, México. 2019. 853p.

[29] García-Bocanegra I, Arenas-Montes A, Hernández E, Adaszek Ł, Carbonero A, Almería S, et al. Seroprevalence and risk factors associated with Babesia caballi and Theileria equi infection in equids. Vet J. 2013; 195, 172–178. doi: 10.1016/j.tvjl.2012.06.012 22784418

[30] Rosales R, Rangel-Rivas A, Escalona A, Jordan LS, Gonzatti MI, Aso PM, et al. Detection of *Theileria equi* and *Babesia caballi* infections in Venezuelan horses using Competitive-Inhibition ELISA and PCR. Vet Parasitol. 2013; 196, 37–43. doi: 10.1016/j.vetpar.2013.02.004 23582233

[31] Jaramillo-Arango CJ, Martínez-Maya JJ. Epidemiología Veterinaria. El Manual Moderno. México D.F. 2010. p. 112–117.

[32] Ueti MW, Palmer GH, Kappmeyer LS, Scoles GA, Knowles DP. Expression of equi merozoite antigen 2 during development of *Babesia equi* in the midgut and salivary gland of the vector tick *Boophlius microplus*. J Clin Microbiol. 2003; 41(12):5803–5809. doi: 10.1128/JCM.41.12.5803-5809.2003 14662988 PMC308990

[33] Ueti MW, Mealey RH, Kappmeyer LS, White SN, Kumpula-McWhirter N, Pelzel AM, et al. Re-Emergence of the Apicomplexan Theileria equi in the United States: Elimination of Persistent Infection and Transmission Risk. PLoS ONE. 2012; 7(9): e44713. doi: 10.1371/journal.pone.0044713 22970295 PMC3435266

[34] Rapoport A, Aharonson-Raz K, Berlin D, Tal S, Gottlieb Y, Klement E, et al. Molecular characterization of the *Babesia caballi* rap-1 gene and epidemiological survey in horses in Israel. Infect Genet Evol. 2014; 23, 115–120. ISSN 1567-1348. doi: 10.1016/j.meegid.2014.01.033 24524984

[35] Wise LN, Pelzel-McCluskey AM, Mealey RH, Knowles DP. Equine Piroplasmosis. Vet Clin Equine. 2014; 30, 677–693. doi: 10.1016/j.cveq.2014.08.008 25300637

[36] Knowles DP, Perryman LE, Goff WL, Miller CD, Harrington RD, Gorham JR. A monoclonal antibody defines a geographically conserved surface protein epitope of *Babesia equi* merozoites. Infect Immun. 1991; 59, 2412–2417. doi: 10.1128/iai.59.7.2412-2417.1991 1711016 PMC258026

[37] Kappmeyer LS, Perryman LE, Hines SA, Baszler TV, Katz JB, Hennager SG, et al. Detection of equine antibodies to *Babesia caballi* by recombinant *B*. *caballi* rhoptry-associated protein 1 in a competitive-inhibition Enzyme-Linked Immunosorbent Assay. J Clin Microbiol. 1999; 37:7, 2285–2290. doi: 10.1128/JCM.37.7.2285-2290.1999 10364599 PMC85139

[38] Ueti MW, Palmer GH, Scoles GA, Kappmeyer LS, Knowles DP. Persistently Infected Horses Are Reservoirs for Intrastadial Tick-Borne Transmission of the Apicomplexan Parasite *Babesia equi*. Infect Immun. 2008; Aug, 3525–3529. doi: 10.1128/IAI.00251-08 18490466 PMC2493223

[39] Mahmoud MS, Abu El-Ezz NT, Abdel-Shafy S, Nassar SA, El-Namaky AH, Khalil WKB, et al. Assessment of Theileria equi and Babesia caballi infections in equine populations in Egypt by molecular, serological and hematological approaches. Parasites Vectors. 2016; 9:260. doi: 10.1186/s13071-016-1539-9 27146413 PMC4857240

[40] Grause JF, Ueti MW, Nelson JT, Knowles DP, Kappmeyer LS, Bunn TO. Efficacy of imidocarb dipropionate in eliminating Theileria equi from experimentally infected horses. Vet J. 2013; 196, 541–546. doi: 10.1016/j.tvjl.2012.10.025 23199699

